# 2-Hydroxy-*N*-phenylbenzamides and Their Esters Inhibit Acetylcholinesterase and Butyrylcholinesterase

**DOI:** 10.3390/biom9110698

**Published:** 2019-11-05

**Authors:** Martin Krátký, Šárka Štěpánková, Neto-Honorius Houngbedji, Rudolf Vosátka, Katarína Vorčáková, Jarmila Vinšová

**Affiliations:** 1 Department of Organic and Bioorganic Chemistry, Faculty of Pharmacy in Hradec Králové, Charles University, Akademika Heyrovského 1203, 500 05 Hradec Králové, Czech Republic; houngben@faf.cuni.cz (N.-H.H.); vosatkar@faf.cuni.cz (R.V.); vinsova@faf.cuni.cz (J.V.); 2 Department of Biological and Biochemical Sciences, Faculty of Chemical Technology, University of Pardubice, Studentská 573, 532 10 Pardubice, Czech Republic; Sarka.Stepankova@upce.cz (Š.Š.); katarina.vorcakova@upce.cz (K.V.)

**Keywords:** acetylcholinesterase, benzamides, butyrylcholinesterase, enzyme inhibition, esters, in vitro inhibition, phosphorus derivatives, salicylanilides

## Abstract

The development of novel inhibitors of acetylcholinesterase (AChE) and butyrylcholinesterase (BuChE) represents a viable approach to alleviate Alzheimer’s disease. Thirty-six halogenated 2-hydroxy-*N*-phenylbenzamides (salicylanilides) with various substitution patterns and their esters with phosphorus-based acids were synthesized in yields of 72% to 92% and characterized. They were evaluated for in vitro inhibition of AChE from electric eel and BuChE from equine serum using modified Ellman’s spectrophotometric method. The benzamides exhibited a moderate inhibition of AChE with IC_50_ values in a narrow concentration range from 33.1 to 85.8 µM. IC_50_ values for BuChE were higher (53.5–228.4 µM). The majority of derivatives inhibit AChE more efficiently than BuChE and are comparable or superior to rivastigmine—an established cholinesterases inhibitor used in the treatment of Alzheimer’s disease. Phosphorus-based esters especially improved the activity against BuChE with 5-chloro-2-{[4-(trifluoromethyl)phenyl]carbamoyl}phenyl diethyl phosphite **5c** superiority (IC_50_ = 2.4 µM). This derivative was also the most selective inhibitor of BuChE. It caused a mixed inhibition of both cholinesterases and acted as a pseudo-irreversible inhibitor. Several structure-activity relationships were identified, e.g., favouring esters and benzamides obtained from 5-halogenosalicylic acids and polyhalogenated anilines. Both 2-hydroxy-*N*-phenylbenzamides and esters share convenient physicochemical properties for blood-brain-barrier penetration and thus central nervous system delivery.

## 1. Introduction

Alzheimer’s disease (AD) is a chronic neurodegenerative disease and the most common cause of dementia, with heavy social and economic costs and problems, which gradually worsen over a number of years [1,2]. Fifty million people worldwide are living with dementia and the World Alzheimer report 2018 estimates an incensement to more than 152 million cases by 2050 [3]. The disease is associated with a loss of cholinergic neurons in the brain and decreased levels of neurotransmitter acetylcholine (ACh). Enzymes called cholinesterases terminate its action. The brain contains two major forms of cholinesterases: acetylcholinesterase (AChE) and butyrylcholinesterase (BuChE). Both enzymes may have roles in the aetiology and progression of AD beyond regulation of synaptic ACh levels. They differ genetically, structurally and in their kinetics. The enzyme acetylcholinesterase (a serine hydrolase found at neuromuscular junctions and cholinergic brain synapses) is involved in the termination of impulse transmission by rapid hydrolysis of ACh in numerous cholinergic pathways in the central and peripheral nervous systems. Butyrylcholinesterase serves as a co-regulator of AChE activity [4]. While the overall amount of AChE in the brain decreases with the progression of AD, the enzymatic activity of BuChE remains unchanged or even increases and can compensate the loss of AChE [5]. The presence of AChE in the patients’ brains is associated with β-amyloid plaques and neurofibrillary tangles (NFT), which are the hallmarks of the AD pathogenesis. Increased AChE levels have been discovered around amyloid plaques and NFT [6]. Analogously, overexpression of BuChE is involved in the formation of β-amyloid protein and senile plaques. Therefore, inhibiting BuChE activity could prove beneficial as a hypothetically disease-modifying approach to AD; BuChE is considered as an adjunctive therapeutic target in later stages of AD [7].

Based on these facts, the main medicines used in the treatment of AD are AChE inhibitors. In addition to potent enzyme inhibition, these molecules must cross the blood-brain barrier (BBB) to be sufficiently available in the central nervous system (CNS). According to the mode of action, AChE inhibitors are irreversible or reversible. Reversible inhibitors, competitive or non-competitive (such as the current AD drugs donepezil and galantamine) and pseudo-irreversible inhibitor rivastigmine, a unique dual inhibitor of AChE and BuChE [8], have therapeutic applications, while toxic effects are normally associated with irreversible AChE inhibitors such as organophosphate pesticides and nerve warfare agents.

Moreover, inhibitors of cholinesterases can be used in the therapy of other diseases than AD, such as other dementias, sleep behaviour disorder, myasthenia gravis, glaucoma, postoperative ileus, bladder distention and intoxication of anticholinergic agents [9,10,11]. Cholinergic hypothesis was the first comprehensive paradigm to explain the memory deficit and cognitive impairment in AD. After that, the loss of cholinergic neurons has been associated with other neurodegenerative disorders. Later, the cholinergic hypothesis in dementia has lost some interest, in particular because of the limited clinical efficacy of ACh augmentation therapy and the rising highlight of the role of Aβ and tau protein pathology. Recently, the cholinergic hypothesis has been “revisited” due to many reasons [12]. In addition to this approach, other hypotheses have been formulated, e.g., amyloid cascade, hyperphosphorylation of tau protein, oxidative stress, metal ions hypothesis, or inflammatory hypothesis [13].

Current treatment cannot stop the disease from progressing; it can only temporarily improve symptoms or slow down the progress at best. Thus, a worldwide effort is needed to find better ways to treat the Alzheimer’s disease, delay its onset, and prevent it from developing. Despite multiple features of AD pathogenesis and a wide range of proposed potential targets, no better therapeutic interventions than inhibitors of cholinesterases and NMDA-receptor antagonist memantine have been introduced. AChE inhibition may decrease Aβ plaques and NFT formation. Despite moderate clinical efficacy of cholinesterases inhibitors, AChE is one of the most investigated targets for AD [6]. Thus, AChE- and BuChE-oriented inhibitors-related research can still be considered useful. In particular, these dual inhibitors modulating also other pathogenesis pathway(s) are up-to-date.

2-Hydroxy-*N*-phenylbenzamides (salicylanilides or 2-hydroxybenzanilides) are weakly acidic phenolic compounds. Depending on their substitution, they can be divided into different groups, including halogenated (mono-, di- and polyhalogenated compounds) and nonhalogenated derivatives, *O*-esters including carbamates with antibacterial, mainly antimycobacterial and antifungal activity [14,15,16,17,18]. Several halogenated salicylanilides are widely used as veterinary drugs against helminths and ectoparasites [19]. Commercially available closantel, niclosamide, oxyclozanide, rafoxanide and resorantel are also potent against Gram-positive bacteria such as *Staphylococcus aureus*, *Streptococcus pyogenes* or *Propionibacterium acnes* [20]. Niclosamide and *N*-(3,5-dichlorophenyl)-5-chloro-2-hydroxybenzamide exhibited anti-filamentation and anti-biofilm activity against fungus *Candida albicans*, and multidrug-resistant yeast *Candida auris* [21].

We have found salicylanilide *O*-substituted derivatives with significant activity against AChE and BuChE. Diethyl phosphates act as pseudo-irreversible cholinesterases inhibitors [22], *N*-phenethyl [23] and *N*,*N*-diphenyl carbamates [24] produced clearly favourable BuChE inhibition when compared to other analogues. In addition, salicylanilide diethyl thiophosphates were active, especially against AChE [25]—but there is no study dealing with the inhibition property of parent benzamides with free phenolic group. That is why we initiated this systematic study dealing with the halogenated salicylanilides as potential inhibitors of both cholinesterases and their phosphorus analogues. The halogenation ensures sufficient lipophilicity, which should contribute to the passive targeting of CNS. The current study should answer whether parent salicylanilides are able to inhibit AChE and BuChE, how selective and if the intrinsic activity of this scaffold could participate in the inhibition previously described for salicylanilide carbamates and (thio)phosphates [22,23,24,25]. The second goal is focused on the influence of esterification by phosphorus-based acids on enzyme inhibition and selectivity. The compounds with low IC_50_ values and dual inhibition of AChE and BuChE with optimal physicochemical properties to penetrate BBB were aimed at in this study.

## 2. Materials and Methods

### 2.1. Chemistry 

#### 2.1.1. General Methods

All the reagents and solvents were purchased from Sigma-Aldrich (Darmstadt, Germany) or Penta Chemicals (Prague, Czech Republic) and they were used as received. The reactions and the purity of the products were monitored by thin-layer chromatography using a mixture with a ratio of toluene to ethyl acetate of 4:1 or 9:1 (*v*/*v*) as the eluent. Plates were coated with 0.2 mm Merck 60 F254 silica gel (Merck Millipore, Darmstadt, Germany) and were visualized by UV irradiation (254 nm). The melting points were determined on a Büchi Melting Point B-540 apparatus (BÜCHI, Flawil, Switzerland) using open capillaries. The reported values are uncorrected. Infrared spectra were recorded on a FT-IR spectrometer using ATR-Ge method (Nicolet 6700 FT-IR, Thermo Fisher Scientific, Waltham, MA, USA) in the range 650–4000 cm^−1^. The NMR spectra were measured in DMSO-d_6_, CDCl_3_ or acetone-d_6_ at ambient temperature using a Varian V NMR S500 instrument (500 MHz for ^1^H and 126 MHz for ^13^C; Varian Comp. Palo Alto, CA, USA). The chemical shifts δ are given in ppm with respect to tetramethylsilane as an internal standard. The coupling constants (*J*) are reported in Hz. Elemental analysis (C, H, N) was performed on an automatic microanalyser CHNS-O CE instrument (FISONS EA 1110, Milano, Italy). Flash chromatography was performed on a CombiFlash Rf 200 automated chromatograph (Teledyne Isco, Lincoln, NE, USA) using columns filled with Kieselgel 60, 0.040–0.063 mm (Merck, Germany) and a detection wavelength of 280 nm.

#### 2.1.2. Synthesis of 2-Hydroxy-*N*-phenylbenzamides 1–4

The microwave-assisted synthesis of salicylanilides **1**–**4** was described previously by our group (e.g., [26]). Briefly, appropriate salicylic acid (1 mmol) was suspended in 10 mL of chlorobenzene, then substituted aniline (1 mmol) was added, followed by phosphorus trichloride (0.5 mmol). The reaction mixture was irradiated in microwave reactor (530 W, 25 min; MicroSYNTH Milestone Ethos 1600 (Milestone, Bergamo, Italy)). The reaction mixture was filtered while hot and was then let to stand at +4 °C for 12 h. The resulted precipitate was filtered off and crystallized from ethanol/water to obtain pure benzamides.

Some of the compounds **1**–**4** were synthesized and reported previously by our group. For this study, we adopted the salicylanilides from two works: Krátký et al. [26] (compounds **1**, **2a**–**2c**, **2f**–**2k**, **3a**–**3c**, **3f**–**3k**, and **4k**) and Paraskevopoulos et al. [15] (derivatives **2l**, **3l**, **4c**, **4g**, and **4l**). Bromosalicylanilides (**4a**, **4b**, **4f**, **4h**–**4j**) were prepared as synthetic precursors of salicylanilide diethyl phosphates [17].

The identity of the known compounds was established using NMR and IR spectroscopy by the comparison with previously reported data. Additionally, their purity was checked by melting points measurement and elemental analysis.

*5-Chloro-N-(3,5-dichlorophenyl)-2-hydroxybenzamide* (**2d**). White solid; yield 82%; mp 246–248 °C (247–249 °C [27]). IR: 3324 (N-H), 1630 (C=O) cm^−1^. ^1^H-NMR (500 MHz, DMSO): δ 11.54 (1H, s, OH), 10.54 (1H, s, NH), 8.09 (2H, d, *J* = 2.3 Hz, H2′, H6’), 7.85 (1H, d, *J* = 2.7 Hz, H6), 7.45 (1H, dd, *J* = 8.8, 2.7 Hz, H4), 7.37 (1H, t, *J* = 2.3 Hz, H4′), 7.01 (1H, d, *J* = 8.8 Hz, H3). ^13^C NMR (126 MHz, DMSO): δ 165.2, 156.7, 139.8, 133.3, 130.6, 128.7, 124.1, 123.0, 120.3, 120.2, 119.2. Anal. C 49.41, H 2.31, N 4.51%, calculated for C_13_H_8_Cl_3_NO_2_ (316.56), C 49.32, H 2.55, N 4.42%.

*5-Chloro-N-(3,4,5-trichlorophenyl)-2-hydroxybenzamide* (**2e**). White solid; yield 79%; mp 286–288.5 °C (287–290 °C [27]). IR: 3323 (N-H), 1628 (C=O) cm^−1^. ^1^H-NMR (500 MHz, DMSO): δ 11.53 (1H, s, OH), 10.63 (1H, s, NH), 8.04 (2H, s, H2′, H6′), 7.83 (1H, d, *J* = 2.7 Hz, H6), 7.45 (1H, dd, *J* = 8.6, 2.5 Hz, H4), 7.05 (1H, d, *J* = 8.8 Hz, H3). ^13^C NMR (126 MHz, DMSO): δ 165.8, 157.4, 149.8, 139.0, 134.6, 132.2, 129.4, 123.0, 120.7, 119.2, 113.5. Anal. C 44.34, H 1.94, N 4.00%, calculated for C_13_H_7_Cl_4_NO_2_ (351.00), C 44.48, H 2.01, N 3.99%.

*4-Chloro-N-(3,5-dichlorophenyl)-2-hydroxybenzamide* (**3d**). White solid; yield 80%; mp 257–259 °C (255–256 °C [28]). IR: 3310 (N-H), 1605 (C=O) cm^−1^. ^1^H-NMR (500 MHz, DMSO): δ 11.70 (1H, s, OH), 10.49 (1H, s, NH), 7.84–7.78 (3H, m, H6, H2′, H6′), 7.31 (1H, t, *J* = 1.9 Hz, H4′), 7.05–6.99 (2H, m, H3, H5). ^13^C NMR (126 MHz, DMSO): δ 166.2, 159.0, 140.9, 137.7, 133.7, 131.4, 122.3, 120.1, 118.8, 118.2, 116.4. Anal. C 49.60, H 2.41, N 4.70%, calculated for C_13_H_8_Cl_3_NO_2_ (316.56), C 49.32, H 2.55, N 4.42%.

*4-Chloro-N-(3,4,5-trichlorophenyl)-2-hydroxybenzamide* (**3e**). White solid; yield 76%; mp 260–262 °C (261–262 °C [29]). IR (ATR): 3320 (N-H), 1609 (C=O) cm^−1^. ^1^H-NMR (500 MHz, DMSO): δ 11.75 (1H, s, OH), 10.55 (1H, s, NH), 8.03 (2H, s, H2′, H6′), 7.83 (1H, d, *J* = 8.5 Hz, H6), 7.06 (1H, d, *J* = 2.1 Hz, H3), 7.02 (1H, dd, *J* = 8.5, 2.1 Hz, H5). ^13^C NMR (126 MHz, DMSO): δ 165.6, 158.3, 138.6, 136.5, 133.0, 131.4, 120.6, 119.6, 119.2, 116.9, 114.0. Anal. C 44.40, H 1.97, N 4.10%, calculated for C_13_H_7_Cl_4_NO_2_ (351.00), C 44.48, H 2.01, N 3.99%.

*5-Bromo-N-(3,5-dichlorophenyl)-2-hydroxybenzamide* (**4d**). White solid; yield 81%; mp 244–245.5 °C (243–245 °C [28]). IR: 3327 (N-H), 1628 (C=O) cm^−1^. ^1^H-NMR (500 MHz, DMSO): δ 11.47 (1H, s, OH), 10.54 (1H, s, NH), 7.93 (1H, d, *J* = 2.5 Hz, H6), 7.81 (2H, d, *J* = 1.9 Hz, H2′, H6′), 7.56 (1H, dd, *J* = 8.8, 2.6 Hz, H4), 7.33 (1H, t, *J* = 1.9 Hz, H4′), 6.95 (1H, d, *J* = 8.8 Hz, H3). ^13^C NMR (126 MHz, DMSO): δ 165.2, 156.8, 140.8, 136.1, 134.2, 131.6, 123.5, 121.1, 119.6, 118.4, 110.4. Anal. C 43.40, H 2.27, N 3.75%, calculated for C_13_H_8_BrCl_2_NO_2_ (361.02), C 43.25, H 2.23, N 3.88%.

*5-Bromo-N-(3,4,5-trichlorophenyl)-2-hydroxybenzamide* (**4e**). White solid; yield 78%; mp 297–298.5 °C (297–299 °C [28]). IR: 3322 (N-H), 1634 (C=O) cm^−1^. ^1^H-NMR (500 MHz, DMSO): δ 11.46 (1H, s, OH), 10.57 (1H, s, NH), 8.01 (2H, s, H2′, H6′), 7.91 (1H, d, *J* = 2.6 Hz, H6), 7.56 (1H, dd, *J* = 8.8, 2.6 Hz, H4), 6.95 (1H, d, *J* = 8.8 Hz, H3). ^13^C NMR (126 MHz, DMSO): δ 165.2, 156.7, 138.6, 136.2, 133.5, 131.7, 124.4, 121.0, 120.6, 119.6, 110.4. Anal. C 39.41, H 1.91, N 3.55%, calculated for C_13_H_7_BrCl_3_NO_2_ (395.46), C 39.48, H 1.78, N 3.54%.

#### 2.1.3. Synthesis of Phosphorus-Based Derivatives **5**

The syntheses of diethyl phosphate **5a** and thiophosphate **5b** were reported previously by our group [17,25]. It is based on the reaction of appropriate salicylanilide with a mild excess of diethyl chlorophosphate or diethyl chlorothiophosphate in the presence of a tertiary base.

Diethyl phosphite **5c** was obtained by the following procedure: 1 mmol of the salicylanilide **3k** was suspended in 10 mL of dichloromethane (DCM) and treated by triethylamine (1.5 of equivalents). After a complete dissolution, diethyl chlorophosphite was added in one portion (1.5 of equivalents). The reaction mixture was stirred for 24 h at room temperature. Then, it was evaporated to provide a viscous liquid. Ethyl acetate was added, and the insoluble portion was filtered off. The filtrate was washed sequentially by sodium carbonate solution (10%), hydrochloric acid (0.1 M) and saturated brine. The organic phase was dried using sodium sulphate and evaporated to provide a viscous oily liquid, which was purified using flash chromatography (mobile phase composition was with a ratio of ethyl acetate to hexane of 1:3 (*v*/*v*)).

Cyclic analogues **5d** and **5e** were synthesized by modified procedure adopted from [30]. 1 mmol of the salicylanilide **3k** was suspended in 10 mL of dichloromethane (DCM) and treated by triethylamine (3 of equivalents). After a complete dissolution, phenylphosphonic dichloride (or phenylphosphonothioic dichloride) was added in one portion (1.5 of equivalents). The reaction mixture was heated for 4 h and then stirred at room temperature. After 20 h, it was evaporated to dryness. The resulting crystals were suspended in ethyl acetate. The insoluble portion was filtered off and the filtrate was washed sequentially by sodium carbonate solution (10%), hydrochloric acid (0.1 M) and saturated brine. The organic phase was dried using sodium sulphate and the crystallization was initiated by the addition of *n*-hexane. The reaction mixture was stored for 24 h at +4 °C. Then, the resulting precipitate was filtered off and the crystals were recrystallized from ethyl acetate/hexane mixture.

*5-Chloro-2-{[4-(trifluoromethyl)phenyl]carbamoyl}phenyl diethyl phosphite* (**5c**). Brownish viscous oil; yield 28%. IR: 3310 (N-H), 1684 (C=O), 1601, 1323, 1292, 1165, 1118, 1066, 1033, 964, 845, 766, 689 cm^−1^. ^1^H-NMR (500 MHz, acetone-d_6_): δ 9.99 (1H, s, NH), 8.06-8.02 (2H, m, H3′, H5′), 7.80 (1H, dd, *J* = 8.3, 1.1 Hz, H3), 7.76–7.72 (2H, m, H2′, H6′), 7.52–7.51 (1H, m, H6), 7.44 (1H, ddd, *J* = 8.3, 2.0, 0.9 Hz, H4), 4.28-4.20 (4H, m, CH_2_), 1.29 (6H, td, *J* = 7.1 Hz, *J* = 1.1 Hz, CH_3_). ^13^C NMR (126 MHz, CDCl_3_): δ 162.0, 147.5 (d, *J* = 7.1 Hz), 141.3, 138.1 (d, *J* = 2.1 Hz), 132.9 (d, *J* = 1.0 Hz), 126.2 (d, *J* = 1.5 Hz), 126.0 (d, *J* = 1.7 Hz), 126.2 (q, *J* = 3.8 Hz), 126.2 (q, *J* = 32.6 Hz), 124.1 (q, *J* = 270.0 Hz), 121.7 (d, *J* = 3.0 Hz), 119.6, 64.1 (d, *J* = 6.0 Hz), 14.0 (d, *J* = 6.8 Hz). Anal. C 49.68, H 4.29, N 3.09%, calculated for C_18_H_18_ClF_3_NO_4_P (435.76), C 49.61, H 4.16, N 3.21%.

*(RS)-7-Chloro-2-phenyl-3-[4-(trifluoromethyl)phenyl]-3-hydrobenzo*[*e*][1,3,2]oxazaphosphinin-4-one 2-oxide (**5d**). White solid; yield 76%; mp 185–186 °C. IR: 3337, 1656 (C=O), 1575, 1564, 1330, 1219, 1181, 1118, 1070, 715 cm^−1^. ^1^H-NMR (500 MHz, CDCl_3_): δ 8.16 (1H, d, *J* = 8.5 Hz, H5), 7.77–7.72 (2H, m, H3′, H5′), 7.66–7.56 (3H, m, H2′, H6′, H4′′), 7.50-7.44 (2H, m, H3′′, H5′′), 7.39-7.28 (4H, m, H6, H8, H2′′, H6′′). ^13^C NMR (126 MHz, CDCl_3_): δ 161.1 (d, *J* = 4.3 Hz), 151.3 (d, *J* = 7.9 Hz), 141.9, 136.6, 134.4 (d, *J* = 3.2 Hz), 132.3 (d, *J* = 11.2 Hz), 131.5, 131.0 (q, *J* = 32.7 Hz), 130.2 (d, *J* = 2.4 Hz), 129.0 (d, *J* = 16.1 Hz), 126.5 (q, *J* = 3.9 Hz), 125.9, 125.5 (d, *J* = 182.6 Hz), 123.5 (q, *J* = 272.6 Hz), 119.4 (d, *J* = 9.2 Hz), 115.8 (d, *J* = 2.0 Hz). Anal. C 54.65, H 2.69, N 3.29%, calculated for C_20_H_12_ClF_3_NO_3_P (437.74), C 54.88, H 2.76, N 3.20%.

*(RS)-7-Chloro-2-phenyl-3-[4-(trifluoromethyl)phenyl]-3-hydrobenzo*[*e*][1,3,2]oxazaphosphinin-4-one 2-sulfide (**5e**). Yellow solid; yield 52%; mp 136.5–139 °C. IR: 3076, 1675 (C=O), 1610, 1417, 1330, 1315, 1299, 1130, 1069, 949, 750, 692 cm^−1^. ^1^H-NMR (500 MHz, CDCl_3_): δ 8.13 (1H, d, *J* = 8.4 Hz, H5), 7.94-7.88 (2H, m, H3′, H5′), 7.64–7.59 (1H, m, H4′′), 7.53 (2H, d, *J* = 8.3 Hz, H2′, H6′), 7.50–7.44 (2H, m, H3′′, H5′′), 7.34 (1H, td, *J* = 8.5, 1.3 Hz, H6), 7.30–7.24 (3H, m, H8, H2′′, H6′′). ^13^C NMR (126 MHz, CDCl_3_): δ 161.3 (d, *J* = 3.7 Hz), 151.5 (d, *J* = 8.7 Hz), 141.7, 134.4 (d, *J* = 3.3 Hz), 132.2 (d, *J* = 12.9 Hz), 131.4, 130.6 (d, *J* = 2.4 Hz), 130.8 (q, *J* = 32.6 Hz), 129.6 (d, *J* = 139.3 Hz), 128.7 (d, *J* = 15.8 Hz), 126.1 (q, *J* = 3.8 Hz), 125.8, 123.5 (q, *J* = 272.5 Hz), 120.9, 119.9 (d, *J* = 8.4 Hz), 116.6 (d, *J* = 3.3 Hz). Anal. C 53.11, H 2.77, N 2.94%, calculated for C_20_H_12_ClF_3_NO_2_PS (453.80), C 52.94, H 2.67, N 3.09%.

#### 2.1.4. Determination of Physicochemical Parameters

The molecular weights, log*P* values and topological polar surface areas (tPSA) were calculated using the CS ChemOffice Ultra program (version 18.0, CambridgeSoft, Cambridge, MA, USA).

### 2.2. Determination of Cholinesterases Inhibition

The IC_50_ values were determined using the spectrophotometric Ellman’s method modified according to Zdražilová et al. [31], which is a simple, rapid and direct method to determine the SH and -S-S- group content in proteins. This method is widely used for the screening of the efficiency of cholinesterases inhibitors. The enzymatic activity is measured indirectly by quantifying the concentration of the 5-thio-2-nitrobenzoic acid ion formed in the reaction between the thiol reagent 5,5′-dithio-bis(2-nitrobenzoic acid) and thiocholine, a product of substrate hydrolysis (i.e., acetylthiocholine; ATCH) by cholinesterases [32].

The enzyme activity in final reaction mixture (2000 µL) was 0.2 U/mL, concentration of acetylthiocholine (or butyrylthiocholine, BTCH) 40 µM and concentration of 5,5′-dithio-bis(2-nitrobenzoic acid) 0.1 mM for all reactions. The tested compounds were dissolved in DMSO and then diluted in demineralized water to the concentration of 1 µM. For all tested compounds and standard (rivastigmine), five different concentrations of inhibitor in final reaction mixture were typically used. All measurements were carried in triplicate and the average values of reaction rate (v_0_-uninhibited reaction, v_i_-inhibited reaction) were used for construction of the dependence v_0_/v_i_ vs. concentration of inhibitor. From obtained equation of regression curve, the value of IC_50_ was calculated (based on the definition of IC_50_).

The determination of mechanism of enzyme inhibition was described in detail previously [22]. The inhibitor **5c** was evaluated at three different concentrations for each enzyme.

For the determination of the type of inhibition Lineweaver-Burk plot [33] was used and the measuring procedure was similar to that for the determination of IC_50_ (modified Ellman’s method was used). The enzyme activity in the final reaction mixture (2000 µL) was 0.2 U/mL, a concentration of ATCH or BTCH 20–80 µM and a concentration of 5,5′-dithio-bis(2-nitrobenzoic acid) 0.1 mM. For each of the substrate concentrations, four different concentrations of inhibitor were chosen. The dependence absorbance vs. time was observed and the reaction rate was calculated. All measurements were carried in duplicate and the average values of reaction rate were used for the construction of the Lineweaver-Burk plot. From obtained equations of regression curves, the values of K_M_ (Michaelis constant) and V_m_ (maximum velocity) were calculated and the type of inhibition was evaluated.

Acetylcholinesterase was obtained from electric eel (*Electrophorus electricus* L.) and butyrylcholinesterase was from an equine serum. Rivastigmine was used as a reference drug. All of the enzymes and rivastigmine were purchased from Sigma-Aldrich (Prague, Czech Republic).

## 3. Results and Discussion

### 3.1. Chemistry

**Scheme 1:**
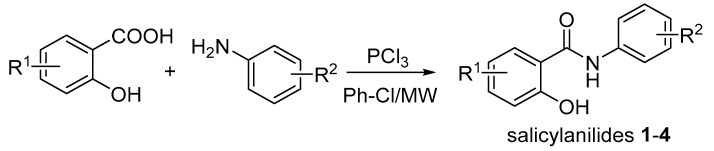
Synthesis of salicylanilides **1**-**4** (R^1^ = H, 5-Cl, 4-Cl, 5-Br; R^2^ = H, 3-F, 3-Cl, 3-Br, 3-CF_3_, 4-F, 4-Cl, 4-Br, 4-CF_3_, 3,4-diCl, 3,5-diCl, 3,4,5-triCl, 3,5-bis-CF_3_; Ph-Cl: chlorobenzene).

2-Hydroxy-*N*-phenylbenzamides **1**–**4l** were synthesized from substituted salicylic acids and anilines using phosphorus trichloride (PCl_3_) under microwave (MW) irradiation (Scheme 1). When compared to analogous reaction performed under refluxing for 6 h, this MW-mediated synthesis offers shorter reaction time and higher yields (72–92%) [26]. The majority of these benzamides have been reported in our previous studies [15,17,26]; for details, see Section 2.1.2.

**Scheme 2:**
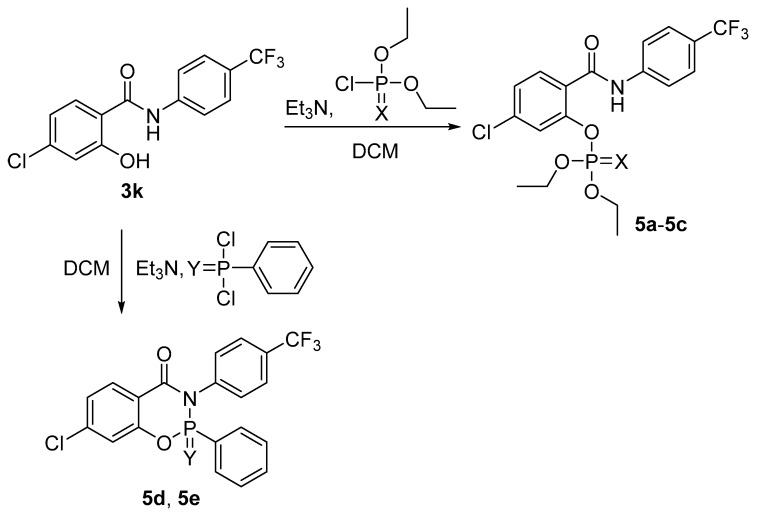
Synthesis of the esters **5** (X = O, S, or is missing; Y = O, S; DCM: dichloromethane).

The selected benzamide **3k** was esterified using corresponding chlorides of phosphorus-based acids. It was converted by triethylamine (Et_3_N; 1.5 of equivalents for **5a**–**5c**, 3 of equivalents for **5d** and **5e**) in situ into triethylammonium salts in dichloromethane (DCM) and then a mild excess (1.5 of equivalents) of chlorides were added [17,25]. After 24 h of stirring, the final esters **5** (Scheme 2) were obtained in yields of 28% to 93%. The preparation of the cyclic analogues **5d** and **5e** involved also initial refluxing for 4 h.

The compounds were characterised by melting points, IR, and NMR spectra. Their purity was checked by thin-layer chromatography and elemental analysis.

The structures of all the compounds are reported in Table 1.

### 3.2. In Vitro Inhibition of Acetylcholinesterase and Butyrylcholinesterase

Thirty-six benzamides **1**–**4l** were evaluated as potential inhibitors of AChE from electric eel (*Ee*AChE) and BuChE from equine serum (EqBuChE) using Ellman’s spectrophotometric method modified according to Zdražilová et al. [31]. Their efficacy is expressed as IC_50_, i.e., the concentration required for 50% inhibition of the enzymatic activity. Based on inhibition of both cholinesterases, we calculated selectivity indexes (SI) as the ratio of IC_50_ value for AChE/IC_50_ value for BuChE to express the selectivity quantitatively (Table 1). The results were compared with those obtained for rivastigmine, an established carbamate drug used in the therapy of Alzheimer’s and Parkinson’s dementias. Rivastigmine is a dual acylating pseudo-irreversible inhibitor of both AChE and BuChE.

Focusing on inhibition of AChE, IC_50_ values were in a close range 33.13 (**4d**)–85.75 (**4b**) μM. These results are fully comparable to those obtained for phenolic carbamate drug rivastigmine (56.10 μM). Interestingly, unsubstituted salicylanilide **1** produced a comparatively better inhibition (49.71 μM) than a majority of salicylanilides, i.e., the halogenation of salicylanilide moiety is not essential for inhibition of AChE. However, the bromination of salicylic acid in combination with a polyhalogenated aniline improved the activity (**1** vs. **4d**, **4e**, **4j**, and **4k**) and provided the three most active compounds in this study, 5-bromo-2-hydroxy-*N*-(3,5-dichlorophenyl)benzamide **4d** (33.13 μM), followed by 5-bromo-2-hydroxy-*N*-(3,4,5-trichlorophenyl)benzamide **4e** (42.08 μM) and 5-bromo-2-hydroxy-*N*-[3-(trifluoromethyl)phenyl]benzamide **4j** (42.67 μM).

Additionally, several general structure-activity relationships were identified:

1. Slightly better inhibition is associated with derivatives of 5-substituted salicylic acid (series **2** and **4**).
2. The substitution of the position 3 at the aniline ring led to improved activity when compared to the position 4 (majority of the series **3** and the pair **2h** and **2i** are partial exceptions).
3. The order of preferred aniline substituents in the case of monosubstitution is as follows: CF_3_ > F > Br > Cl (series **4**); Cl > Br > F > CF_3_ (series **3**); F > Br > CF_3_ > Cl (series **2**).
4. The di- and tri-substitution of aniline (both by chlorine **c**–**e** and trifluoromethyl group **l**) resulted predominantly in significantly more potent inhibition of AChE when compared to monosubstituted analogues.
5. Regarding polychlorinated anilides, the derivatives of 3,5-dichloroaniline **d** are superior to 3,4-dichloroanilides **c** and also to 3,4,5-trichloroanilides **e**; the additional substitution of the 3,5-dichloro compounds **d** by 4-chlorine (i.e., providing trichloro structures **e**) is detrimental but it is still superior to 3,4-dichloroanilides **c**. Series **3** is a partial exception.

In contrast to AChE, somewhat different results were obtained for BuChE. This enzyme was inhibited at higher concentrations and in a broader concentration range starting from 53.46 μM (**4l**) up to 228.42 μM (**2a**); all the compounds are less potent than rivastigmine (38.40 μM). The halogenation of the salicylanilide core is essential and it improves IC_50_ values of the salicylanilide **1**. Two amides share significantly low IC_50_ values: 5-bromo- and 5-chloro-2-hydroxy-*N*-[3,5-bis(trifluoromethyl)phenyl]benzamides **2l** and **4l** (64.44 and 53.46 μM, respectively). The following SAR were revealed:

1. The most potent inhibition was exhibited by the derivatives of 5-bromosalicylic acid (series **4**) followed by 5-chlorosalicylic acid (**2**).
2. The order of preferred atoms on monosubstituted aniline ring is as follows: Br > CF_3_ > F > Cl (series **2**); Cl > F > Br > CF_3_ (series **3**); CF_3_ > F > Br > Cl (series **4**; i.e., the identical results as obtained for AChE). There was no sharp difference in the activity of 3- and 4-substituted anilines.
3. The presence of di- and tri-substituted anilines increases the BuChE inhibition predominantly, especially for trifluoromethyl derivatives.
4. Regarding polychlorinated anilides within the series **3** and **4**, the derivatives of 3,4-dichloroaniline **c** are superior to 3,5-dichloroanilides **d** and also to 3,4,5-trichloroanilides **e**. However, the reverse SAR was found in the series **2**.

Regarding selectivity to both cholinesterases, it can be distinguished based on the values of SI. SI lower than 1 means selectivity for AChE. On the other hand, compounds with SI higher than 1 tends to the selectivity for BuChE. The salicylanilides **1**–**4** share an obvious tendency to inhibit AChE more efficiently (SI within the range of 0.24 to 0.91; Table 1). Only the bis-trifluoromethyl derivative **4l** exhibited almost identical inhibition of both enzymes.

To investigate the influence of phosphorus-based substituents, a known scaffold for the inhibition of cholinesterases [8,22,25], of phenolic group on inhibition, we prepared several derivatives of 4-chloro-2-hydroxy-*N*-[4-(trifluoromethyl)phenyl]benzamide **3k**, i.e., the salicylanilide with a moderate activity against AChE and low activity against BuChE. Thus, if the presence of phosphorus-based moiety was beneficial, a drop in IC_50_ values would be more significant.

Previously, we synthesized diethyl phosphate **5a** [17,22] and diethyl thiophosphate **5b** [25]. Both derivatives share an enhanced inhibition of BuChE (up to 20.3 times) and moreover, thiophosphate **5b** was 6.6 times more effective AChE inhibitor (Table 2). Then, we prepared analogous diethyl phosphite **5c**. Regarding BuChE, this modification was the most successful one with IC_50_ value of 2.37 µM (84.2 times more potent than the parent **3k**) and highly selective for BuChE (SI 28.0), but still remained the dual inhibitor of both cholinesterases. The removal of oxygen from phosphate **5a** to phosphite **5c** improved the activity especially against BuChE (4.2 times). Then, we prepared two cyclic analogues, 7-chloro-2-phenyl-3-[4-(trifluoromethyl)phenyl]-3-hydrobenzo[*e*][1,3,2]oxazaphosphinin-4-one 2-oxide (**5d**)/2-sulfide (**5e**). The first derivative showed an enhanced inhibitory potency against both enzymes when compared to parent salicylanilide and, regarding AChE, also when compared to the majority of the compounds involved in this study (IC_50_ 48.13 µM). However, the isosteric replacement of oxygen by sulphur (**5d**→**5e**) resulted in a substantial lower activity comparable to parent amide **3k**; it contrasts with the pair **5a** and **5b**, where this switch led to a sharply stronger AChE inhibition. The activity of [1,3,2]oxazaphosphinine-4-ones **5d** and **5e** suggests that the amidic hydrogen is not essential for the existence of biological activity, but its presence contributes to more potent enzyme inhibition.

Four phosphorus-based esters (**5a**–**5d**) were superior to rivastigmine against BuChE and two of them against AChE (**5b**, **5d**). Interestingly, the type of phosphorus functional group can modulate the AChE/BuChE selectivity. The presence of only oxygen(s) leads to preferential inhibition of BuChE (SI of 1.93–28.0; **5a**, **5c** and **5d**) while the incorporation of sulphur increased the relative affinity to AChE (SI 0.37 and 0.50 for **5e** and **5b**, respectively).

We have demonstrated unequivocally that both salicylanilides and their phosphorus-based analogues act as dual AChE-BuChE inhibitors. Based on anti-AD drug rivastigmine, it was shown that this dual inhibition might be beneficial for the therapy of dementia [34]. The parent 2-hydroxybenzamides inhibit AChE preferentially and their modification did not improve the activity predominantly. On the other hand, the esterification of phenolic hydroxyl led to a significantly enhanced inhibition of BuChE. Regarding the structure of phosphorus part of the molecules, it is possible to modulate selectivity to each cholinesterase.

#### Mechanism and Type of Inhibition

Previously, we identified salicylanilide diethyl phosphates as pseudo-irreversible inhibitors of both cholinesterases [22]. That is why we also investigated the mechanism of inhibition of the most potent BuChE inhibitor **5c**.

Based on the changes of enzyme activity in the presence of tested inhibitor during prolonged incubation, it is possible to distinguish reversible, pseudo-irreversible and irreversible inhibition [35,36]. In the case of reversible inhibition, the inhibitor is bound to the enzyme for a short period of time, the activity of the enzyme goes down immediately and, after dissociation of complex enzyme-inhibitor, the enzyme activity is restored. During pseudo-irreversible inhibition, the inhibitor is bound to the enzyme molecule covalently, but the bond is slowly broken down and the enzyme activity returns to the initial state. For irreversible inhibition, covalent and permanent bond between enzyme and inhibitor is typical and therefore the enzyme becomes inactivated. Irreversible inhibitor decreases enzyme activity successively and the dependence log% A vs. time in the presence of inhibitor is linear [37,38].

**Figure 1:**
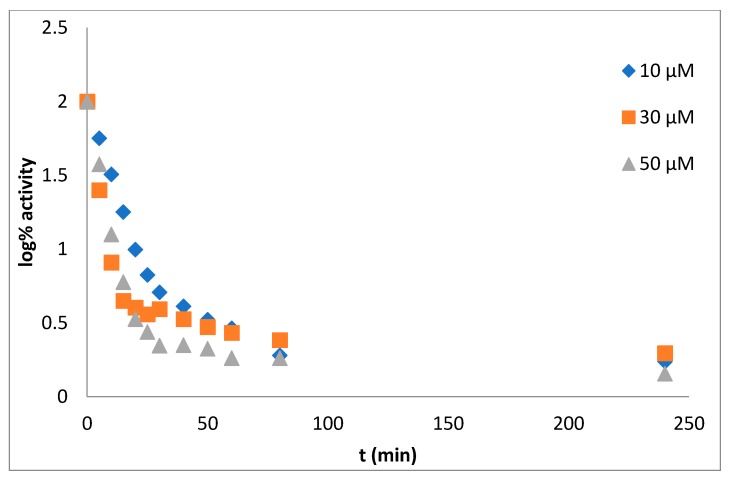
The dependence log% A vs. time. Enzyme: acetylcholinesterase, derivative **5c**, concentration of inhibitor: 10 µM (rhomb), 30 µM (square), 90 µM (triangle).

**Figure 2:**
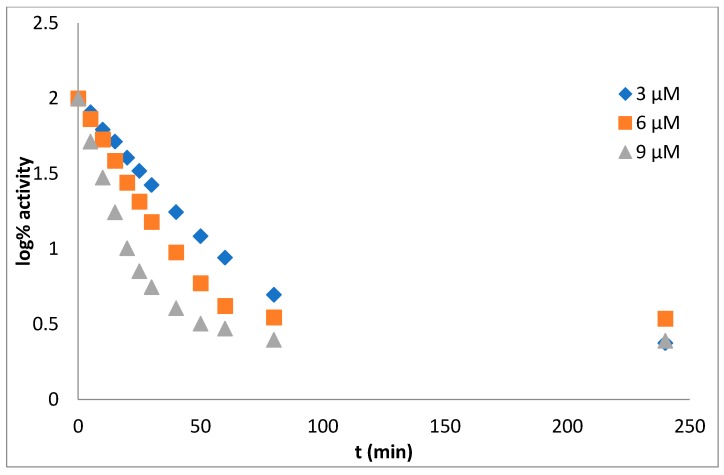
The dependence log% A vs. time. Enzyme: butyrylcholinesterase, derivative **5c**, concentration of inhibitor: 3 µM (rhomb), 6 µM (square), 9 µM (triangle).

The procedure of measurement and data evaluation were in detail described previously in the reference [22]. From the dependence log% A vs. time the mechanism of action of tested inhibitor can be deduced. Obtained results assert the pseudo-irreversible inhibition of both cholinesterase enzymes (Figure 1 for AChE, Figure 2 for BuChE).

The enzyme inhibitors may bind to the active site or at some other sites. Based on this fact, they can be divided into four categories: competitive, non-competitive, uncompetitive or mixed. Competitive inhibitors compete with the substrate for the same binding site on the enzyme, the so-called active site. Non-competitive, uncompetitive and mixed inhibitors do not bind at the active site but at some other [39].

The type of inhibition could be distinguished based on the Lineweaver-Burk plot [33] and the comparison of the kinetic parameters K_M_ and V_m_ of uninhibited and inhibited reactions. The diagnostic criterion for competitive inhibition is that V_m_ is unaffected by inhibitor and K_M_ for inhibited reaction is increased. All lines in Lineweaver-Burk plot share a common *y*-intercept. For non-competitive inhibitor K_M_ is unchanged, but V_m_ of inhibited reaction is decreased. All lines in Lineweaver-Burk plot share a common *x*-intercept. Uncompetitive inhibitor decreases K_M_ and V_m_, but K_M_/V_m_ remains the same as for the uninhibited reaction. The pattern obtained in Lineweaver-Burk plot is a set of parallel lines. For mixed inhibition K_M_, V_m_ and K_M_/V_m_ are altered. The lines in Lineweaver-Burk plot intercept in the quadrant II or III [33].

**Figure 3:**
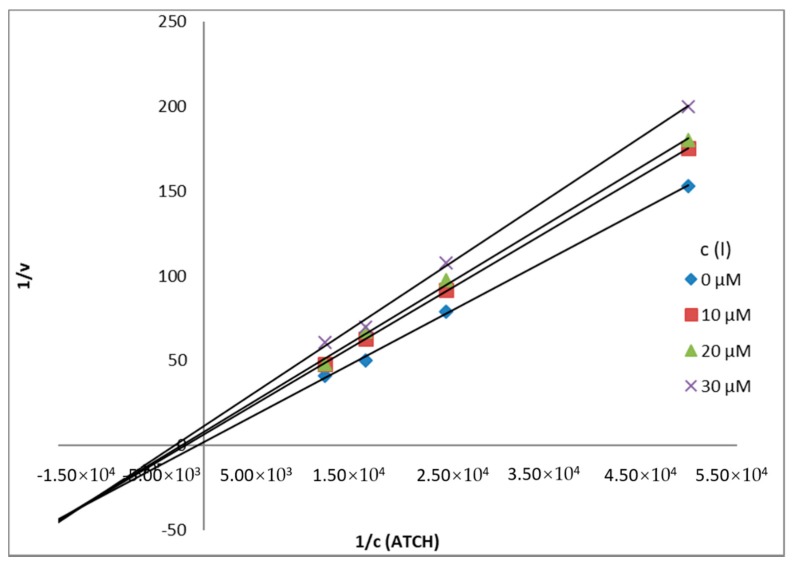
Lineweaver-Burk plot for AChE inhibition (ATCH = acetylthiocholine).

**Figure 4:**
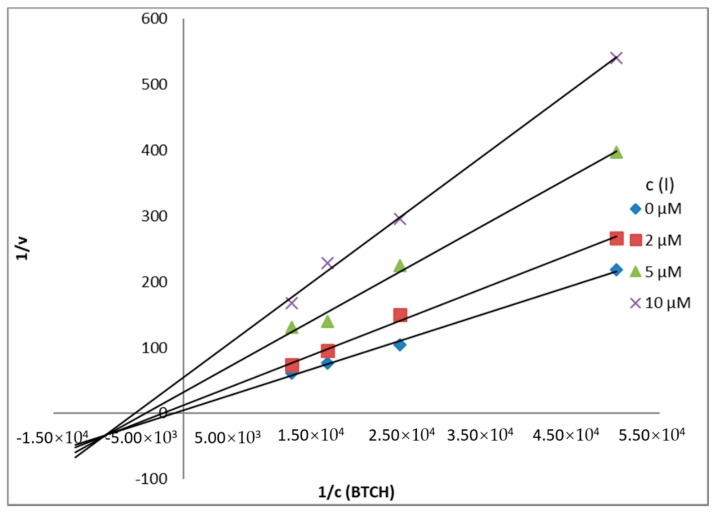
Lineweaver-Burk plot for BuChE inhibition (BTCH = butyrylthiocholine).

From obtained results of K_M_ and V_m_ and Lineweaver-Burk plots presented in Figure 3 (AChE) and Figure 4 (BuChE), it is possible to conclude, that the inhibitor **5c** acts via mixed inhibitory mechanism.

### 3.3. Prediction of Physicochemical Parameters, Drug-Likeness and CNS Delivery

We determined physicochemical parameters that are important for the prediction of drug-likeness and also CNS availability (Table 3). Lipinski’s rule of five defines four physicochemical parameters that are associated with a favorable profile for oral administration. It postulates that the molecular weight (MW) should not be higher than 500, log*P* over five, number of hydrogen bond donors and acceptors should be of ≤5 and ≤10, respectively [40]. All of the derivatives **1**–**5** meet the criteria for MW and the number of hydrogen bond donors and acceptors. One salicylanilide (**4l**) and four phosphoric derivatives (**5a**, **5b**, **5d**, and **5e**) share slightly higher log*P* values. However, the escalated lipophilicity may help to overcome bioavailability-related problems of the parent compounds **1**–**4**, which is known substantially low for salicylanilides, despite generally meeting Lipinski’s rule [14,41]. Increased lipophilicity should also improve crossing biological barriers including BBB by a non-specific passive diffusion.

Polar surface area (PSA) was described as another crucial factor for drugs targeting CNS. PSA is calculated as a sum of polar atoms surfaces (i.e., nitrogens, oxygens and their attached hydrogens) [42]. A PSA value of less than 76 Å^2^ should indicate an increased probability of BBB permeability [43]. Table 3 reports topological polar surface areas (tPSA) calculated using ChemOffice software. All the investigated derivatives exhibited a sufficiently low tPSA values.

Ghose et al. [43] analysed a range of CNS and non-CNS drugs and, in addition to tPSA, they provided additional parameters for designing CNS drugs including: (i) ideally one or two nitrogen atom(s), (ii) fewer than seven (two to four) linear chains outside of rings, (iii) fewer than three (zero or one are optimal) polar hydrogens. Obviously, our compounds meet these guidelines.

## 4. Conclusions

We evaluated 36 lipophilic halogenated 2-hydroxy-*N*-phenylbenzamides **1**–**4** prepared using PCl_3_ under MW conditions in high yields. The selected salicylanilide was esterified by chlorides of phosphorus-based acids in the presence of a tertiary base. These derivatives were screened in vitro for their activity against acetylcholinesterase and butyrylcholinesterase.

All salicylanilides exhibited moderate inhibition of both cholinesterases in micromolar concentration range starting from 33.1 μM with a clear tendency to affect AChE at lower concentrations and in a closer range of IC_50_. Some of them were superior to phenolic carbamate rivastigmine against this enzyme. Structure-activity relationships were also identified. Introduction of a phosphorus-based acid fragment (monovalent or bivalent) improved inhibitory potency for BuChE significantly uniformly. Importantly, depending of type of phosphorus containing moiety, it is possible to modulate the selectivity to cholinesterases. For the most active BuChE inhibitor, type and mechanism of inhibition were determined experimentally. Based on in silico prediction/calculation, the investigated compounds meet the criteria for drug-likeness and various parameters for BBB permeability/CNS availability.

Parent 2-hydroxy-*N*-phenylbenzamides (salicylanilides) with free phenolic group can be considered as convenient compounds (with an intrinsic activity especially against AChE and good physicochemical properties for CNS delivery) for conjugation with another cholinesterase inhibiting scaffolds to obtain drugs that are more active.

## Figures and Tables

**Table 1:** Structures of 2-hydroxybenzamides and IC_50_ for acetylcholinesterase and butyrylcholinesterase.

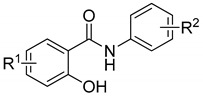					
Code	R^1^	R^2^	IC_50_ (*Ee*AChE) [µM]	IC_50_ (EqBuChE) [µM]	Selectivity AChE/BuChE
**1**	H	H	49.71 ± 3.73	205.73 ± 12.15	0.24
**2a**	5-Cl	3-Cl	59.50 ± 3.80	228.42 ± 11.29	0.26
**2b**	5-Cl	4-Cl	76.53 ± 2.17	169.60 ± 3.09	0.45
**2c**	5-Cl	3,4-diCl	60.79 ± 6.88	186.47 ± 15.69	0.33
**2d**	5-Cl	3,5-diCl	46.21 ± 0.63	111.59 ± 7.98	0.41
**2e**	5-Cl	3,4,5-triCl	51.06 ± 0.49	102.72 ± 0.97	0.50
**2f**	5-Cl	3-Br	50.03 ± 0.15	141.04 ± 1.13	0.35
**2g**	5-Cl	4-Br	58.25 ± 4.87	118.74 ± 6.65	0.49
**2h**	5-Cl	3-F	58.65 ± 3.98	154.40 ± 8.38	0.38
**2i**	5-Cl	4-F	48.27 ± 8.90	132.99 ± 15.81	0.36
**2j**	5-Cl	3-CF_3_	54.41 ± 0.25	120.30 ± 3.02	0.45
**2k**	5-Cl	4-CF_3_	58.47 ± 2.80	154.65 ± 3.58	0.38
**2l**	5-Cl	3,5-bis-CF_3_	50.18 ± 0.88	**64.44 ± 1.34**	0.78
**3a**	4-Cl	3-Cl	58.08 ± 1.20	134.29 ± 0.24	0.43
**3b**	4-Cl	4-Cl	56.19 ± 3.23	152.00 ± 3.13	0.37
**3c**	4-Cl	3,4-diCl	46.26 ± 0.56	100.27 ± 1.15	0.46
**3d**	4-Cl	3,5-diCl	50.15 ± 0.26	141.10 ± 2.80	0.36
**3e**	4-Cl	3,4,5-triCl	57.78 ± 4.05	132.36 ± 4.31	0.44
**3f**	4-Cl	3-Br	60.03 ± 0.42	167.80 ± 2.06	0.36
**3g**	4-Cl	4-Br	58.32 ± 1.57	173.99 ± 4.02	0.34
**3h**	4-Cl	3-F	60.79 ± 1.74	145.66 ± 4.07	0.42
**3i**	4-Cl	4-F	62.85 ± 1.53	151.76 ± 7.58	0.41
**3j**	4-Cl	3-CF_3_	68.28 ± 3.78	163.18 ± 11.68	0.42
**3k**	4-Cl	4-CF_3_	60.29 ± 2.39	199.53 ± 2.02	0.30
**3l**	4-Cl	3,5-bis-CF_3_	54.72 ± 2.05	150.50 ± 0.87	0.36
**4a**	5-Br	3-Cl	62.04 ± 6.48	134.00 ± 1.63	0.46
**4b**	5-Br	4-Cl	85.75 ± 6.10	130.61 ± 3.99	0.66
**4c**	5-Br	3,4-diCl	60.83 ± 3.65	122.60 ± 7.20	0.50
**4d**	5-Br	3,5-diCl	**33.13 ± 0.47**	135.92 ± 0.14	0.24
**4e**	5-Br	3,4,5-triCl	**42.08 ± 2.41**	140.07 ± 6.20	0.30
**4f**	5-Br	3-Br	69.72 ± 9.97	145.78 ± 3.53	0.48
**4g**	5-Br	4-Br	70.30 ± 2.85	112.16 ± 1.79	0.63
**4h**	5-Br	3-F	54.44 ± 0.73	119.24 ± 2.53	0.46
**4i**	5-Br	4-F	61.22 ± 1.08	136.57 ± 1.47	0.45
**4j**	5-Br	3-CF_3_	**42.67 ± 0.18**	116.91 ± 2.23	0.36
**4k**	5-Br	4-CF_3_	44.79 ± 3.44	134.81 ± 0.86	0.33
**4l**	5-Br	3,5-bis-CF_3_	48.46 ± 1.07	**53.46 ± 3.20**	0.91
Rivastigmine			56.10 ± 1.41	38.40 ± 1.97	1.46

AChE and BuChE inhibition are expressed as the mean ± SD (*n* = three experiments). The two or three lowest IC_50_ values for each enzyme are shown in bold.

**Table 2:** Phosphorus-bases inhibitors of AChE and BuChE.

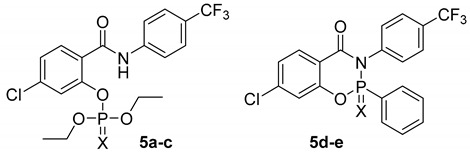				
Code	X	IC_50_ (*Ee*AChE) [µM]	IC_50_ (EqBuChE) [µM]	Selectivity AChE/BuChE
**5a** [22]	O	86.3 ± 4.9	9.84 ± 0.06	8.77
**5b** [25]	S	**9.16 ± 0.17**	18.3 ± 1.0	0.50
**5c**	−	66.37 ± 1.14	**2.37 ± 0.01**	28.00
**5d**	O	48.13 ± 1.70	25.00 ± 0.30	1.93
**5e**	S	63.48 ± 1.41	170.10 ± 13.58	0.37
**3k**		60.29 ± 2.39	199.53 ± 2.02	0.30
Rivastigmine		56.10 ± 1.41	38.40 ± 1.97	1.46

AChE and BuChE inhibition are expressed as the mean ± SD (*n* = three experiments). The lowest IC_50_ value for each enzyme is shown in bold.

**Table 3:** Lipinski’s rule of five parameters and PSA values of the derivatives **1**-**5.**

Code	R^1^	R^2^	MW	Log*P*	H-bond Donors	H-bond Acceptors	Number of Violations	tPSA [Å^2^]
**1**	H	H	213.24	2.45	2	3	0	49.33
**2a**	5-Cl	3-Cl	282.12	3.57	2	3	0	49.33
**2b**	5-Cl	4-Cl	282.12	3.57	2	3	0	49.33
**2c**	5-Cl	3,4-diCl	316.56	4.12	2	3	0	49.33
**2d**	5-Cl	3,5-diCl	316.56	4.12	2	3	0	49.33
**2e**	5-Cl	3,4,5-triCl	351.00	4.68	2	3	0	49.33
**2f**	5-Cl	3-Br	326.57	3.84	2	3	0	49.33
**2g**	5-Cl	4-Br	326.57	3.84	2	3	0	49.33
**2h**	5-Cl	3-F	265.67	3.17	2	3	0	49.33
**2i**	5-Cl	4-F	265.67	3.17	2	3	0	49.33
**2j**	5-Cl	3-CF_3_	315.68	3.93	2	3	0	49.33
**2k**	5-Cl	4-CF_3_	315.68	3.93	2	3	0	49.33
**2l**	5-Cl	3,5-bis-CF_3_	383.67	4.85	2	3	0	49.33
**3a**	4-Cl	3-Cl	282.12	3.57	2	3	0	49.33
**3b**	4-Cl	4-Cl	282.12	3.57	2	3	0	49.33
**3c**	4-Cl	3,4-diCl	316.56	4.12	2	3	0	49.33
**3d**	4-Cl	3,5-diCl	316.56	4.12	2	3	0	49.33
**3e**	4-Cl	3,4,5-triCl	351.00	4.68	2	3	0	49.33
**3f**	4-Cl	3-Br	326.57	3.84	2	3	0	49.33
**3g**	4-Cl	4-Br	326.57	3.84	2	3	0	49.33
**3h**	4-Cl	3-F	265.67	3.17	2	3	0	49.33
**3i**	4-Cl	4-F	265.67	3.17	2	3	0	49.33
**3j**	4-Cl	3-CF_3_	315.68	3.93	2	3	0	49.33
**3k**	4-Cl	4-CF_3_	315.68	3.93	2	3	0	49.33
**3l**	4-Cl	3,5-bis-CF_3_	383.67	4.85	2	3	0	49.33
**4a**	5-Br	3-Cl	326.57	3.84	2	3	0	49.33
**4b**	5-Br	4-Cl	326.57	3.84	2	3	0	49.33
**4c**	5-Br	3,4-diCl	361.02	4.4	2	3	0	49.33
**4d**	5-Br	3,5-diCl	361.02	4.4	2	3	0	49.33
**4e**	5-Br	3,4,5-triCl	395.46	4.95	2	3	0	49.33
**4f**	5-Br	3-Br	371.03	4.11	2	3	0	49.33
**4g**	5-Br	4-Br	371.03	4.11	2	3	0	49.33
**4h**	5-Br	3-F	310.12	3.44	2	3	0	49.33
**4i**	5-Br	4-F	310.12	3.44	2	3	0	49.33
**4j**	5-Br	3-CF_3_	360.13	4.2	2	3	0	49.33
**4k**	5-Br	4-CF_3_	360.13	4.2	2	3	0	49.33
**4l**	5-Br	3,5-bis-CF_3_	428.13	5.12	2	3	1	49.33
**5a**	-	-	451.76	5.08	1	6	1	73.86
**5b**	-	-	467.82	5.82	1	5	1	56.79
**5c**	-	-	435.76	4.70	1	5	0	56.79
**5d**	-	-	437.74	5.64	0	4	1	46.61
**5e**	-	-	453.80	6.37	0	3	1	29.54
